# Evolving Public Attitudes Towards the HPV Vaccine in China: A Fine-Grained Emotion Analysis of Sina Weibo (2016 vs. 2024)

**DOI:** 10.3390/e27090887

**Published:** 2025-08-22

**Authors:** Bowen Shi, Ruibo Chen, Xinyue Yuan, Junran Wu

**Affiliations:** 1 School of Journalism, Communication University of China, Beijing 100024, China; bowenshi@cuc.edu.cn (B.S.); yuanxy@cuc.edu.cn (X.Y.); 2 State Key Laboratory of Software Development Environment, Beihang University, Beijing 100191, China; chenruibo@buaa.edu.cn; 3 School of Computing, National University of Singapore, Singapore 117417, Singapore

**Keywords:** structural entropy, emotion classification, HPV vaccine, health communication, social media

## Abstract

In the digital age, social media significantly shapes public attitudes and emotional responses towards health interventions, such as HPV vaccination, which is critical in developing countries. This study employed a deep learning model to identify fine-grained emotions of 38,615 HPV-related tweets from 2016 to 2024, revealing significant shifts in public emotions. Notably, skepticism about vaccine commercialization motives heightened anger, while university outreach initiatives fostered positive emotions. Structural entropy analysis highlighted polarized emotional communication networks: the network of joy exhibited lower entropy with centralized information flow, whereas other emotions displayed higher entropy, fragmented dissemination, and enhanced cross-community communication efficiency. New communicators, such as campus accounts and music bloggers, played pivotal roles in spreading positive emotions, while individual bloggers in specific fields amplified negative emotions like anger, particularly in closed networks. This research underscores the intricate dynamics of online health communication and the need for targeted interventions to address stigma and enhance public awareness of HPV vaccination, providing valuable insights for future public health policy.

## 1. Introduction

Human papillomavirus (HPV) causes almost all cervical cancer cases and significantly contributes to other anogenital cancers as well as head and neck cancers [1,2]. Cervical cancer ranks as the fourth most common cancer among women globally, and the HPV vaccine can reduce its incidence by approximately 70% [3]. However, in some developing countries with inadequate vaccine coverage [4], the HPV vaccine is relatively new to their populations, and public attitudes may evolve alongside growing awareness and policy updates. In the largest developing country, the HPV vaccine was approved by the China Food and Drug Administration (CFDA) in July 2016. Although China’s HPV vaccination rate rose from 0.30% in 2018 to 2.24% in 2020 [5], it remains well below the World Health Organization targets [6]. By the end of 2024, public opinion erupted over various issues, including sluggish sales of the HPV vaccine, stigmatization, and encouragement for male vaccination, which have sparked a variety of emotional reactions from the public. Research has revealed that exposure to vaccine-related information could affect public perception and vaccination willingness [7]. Thus, monitoring the evolution of public attitudes and emotional responses towards HPV vaccines is critical for strengthening public health and social governance in developing countries.

In today’s digital age, individuals increasingly rely on the Internet for health information, with social media emerging as a crucial platform for disseminating such content [8]. Prior studies indicate that vaccine-related information on social media significantly influences public perceptions. Some scholars found that anti-vaccine messages disseminated by bots on Twitter (X) heighten vaccine skepticism [9]. On Facebook, Luisi suggested that negative emotion predominated in HPV vaccine-related tweets [10], frequently contributing to vaccine hesitancy, and the temporal dynamics of these tweets further showed that negative content facilitates the dissemination of anti-vaccine emotion. Notably, digital emotion contagion has been proven to be widespread on online social media [11], and its aggregated fine-grained emotions are comprehensive indicators of mental health and social risk, which cannot be simplified [12,13]. For instance, sadness is a core component of depression [14], anger can trigger protest movements [13,15], and fear-based sensitivity to threats may provoke widespread public panic [16]. Existing research has mainly explored the impact of health messages on audience behaviors or strategies to increase vaccination intention [7,17,18], as well as the binary emotional polarity associated with vaccines [10,19,20,21]. However, an in-depth analysis of the evolution of public fine-grained emotions and concerns is lacking.

As one of the world’s largest markets for HPV vaccines, the attitudes and emotions of the Chinese public toward HPV vaccination can offer valuable insights with global relevance. This study aims to mine data from Chinese social media platforms and then use complex network and text mining techniques to analyze the evolution of the Chinese public’s fine-grained emotions towards HPV. In contrast to traditional methodologies including interviews and surveys [22], social media, with extensive digital footprints from vast user bases, provides a more effective but less costly proxy for examining users’ authentic attitudes and their evolution over time. Natural language processing technology can automatically identify emerging themes and emotional tendencies from massive unstructured public discourse and can be combined with network analysis to capture the structural evolution of emotional dissemination, reflecting the adaptability and scalability in understanding large-scale complex datasets. In particular, Sina Weibo, a prominent social media platform in China [23], had 523 million monthly active users [24]. Considering that the public online discussions of health issues are typically event-driven [25], this study selected the most significant HPV-related public opinion events from 2016 and 2024. Based on the life cycle theory [26], some scholars pointed out that the period division of public opinion should be combined with the evolutionary characteristics [27]. Therefore, this study combed through Weibo posts related to the hot events, covering the entire lifecycle of each event from initial occurrence to peak intensity and subsequent decline in public interest. Furthermore, based on Plutchik’s wheel of emotions [28], we employed a deep learning model to classify posts into anger, disgust, joy, sadness, and fear. Through fine-grained emotion classification, topic modeling, and structural entropy analysis of retweeting network analysis, this study conducted a comprehensive comparative analysis of public attitudes towards HPV between 2016 and 2024, providing valuable insights for future public health policy enhancement. The specific research questions are as follows:

**RQ1:**  *What was the Chinese public’s emotional distribution toward the HPV vaccine in 2016 and 2024, respectively?*

**RQ2:**  *What central themes about the HPV vaccine emerged from these emotional evolutions?*

**RQ3:**  *From the perspective of information dissemination, are there any thematic differences in the impacts of super-spreaders on various public emotions? If any, what insights can be gained on public opinion guidance strategies?*

## 2. Methods

### 2.1. Data Collection and Preprocessing

Based on the official Weibo index (data.weibo.com/index, accessed on 3 June 2025), this study identified two highly representative public opinion events related to HPV in 2016 and 2024: the approval of HPV vaccines for sale and unsold HPV vaccines. The transmission peaks of these events were determined to be 14 July 2016, and 13 September 2024, respectively. Throughout the complete lifecycle of these events—from emergence to peak engagement and subsequent decline—this study collected 20,560 and 18,055 tweets in 2016 and 2024, respectively, using an application program interface (API) with the keyword “HPV”. Figures S1 and S2 illustrate the trend of the number of tweets over time for two events, which aligns with the typical lifecycle stages of dormant, outbreak, and decline. Notably, the 2024 event presents two distinct peaks, suggesting secondary waves of information dissemination as described by Zhang et al. [29], such as those arising from in-depth media coverage or retweeted by users from different social circles.

For subsequent emotional classification and topic analysis, the raw data underwent systematic cleaning and preprocessing. Firstly, we removed redundant data and outliers, including posts containing only URLs and deactivated user accounts, using user and post metadata to ensure data quality. Secondly, after word segmentation and removing stop-words, we filtered out posts that were too short or only contained emoticons. Some posts only containing hashtags but unrelated to the topic were identified and removed using methods such as regular expressions and keyword matching. Ultimately, each tweet was saved in JavaScript Object Notation (JSON) file, including text, geographic location, retweeting status, and user demographics such as verified type, gender, number of followers, etc.

Unlike X, Weibo mandates real-name registration for all ordinary user and grants red or blue “V” badges to specific verified users, commonly referred to as “Big Vs” [30]. This type of user mainly includes: (1) reputable individuals influential in specific fields; (2) well-known enterprises and their executives; (3) campuses or organizations; (4) mainstream media; and (5) government agencies and other public sectors. It is noteworthy that at the end of 2017, Weibo launched the “V+Membership” service, enabling individual bloggers to provide paid content such as articles and live broadcasts. This service also introduced topic tags that facilitated subsequent user segmentation. To enhance content discoverability, the platform aggregates topics and assigns tags (e.g., “Entertainment”, “Makeup”, and “Games”) to bloggers based on their vertical contents. In subsequent functional iterations, the platform continuously refines its classification system to improve content distribution efficiency and optimize monetization model.

### 2.2. Emotion Classification

Emotional analysis of user posts on social media reveals underlying public attitudes, particularly the dynamic interplay of various negative emotions and resultant tensions, which profoundly influence public health [31]. Based on Plutchik’s Emotion Wheel [28], this study integrated multiple datasets with fine-grained emotion annotations, including NLPCC13, NLPCC14, and SMP2020 (data available in http://tcci.ccf.org.cn/conference/2013/, http://tcci.ccf.org.cn/conference/2014/, https://smp2020ewect.github.io/, accessed on 3 June 2025), ultimately constructing a corpus of over 50,000 texts. To ensure balanced training data across categories, we randomly sampled 3000 texts per emotion category from multi-source labeled Weibo datasets. Then a deep learning model was trained to classify these texts into five basic emotions: anger, disgust, happiness, sadness and fear. In contrast to commonly used deep learning models such as BiLSTM [23,32], BERT excelled in text classification tasks due to its bidirectional masked language model [33]. RoBERTa, an optimized iteration of BERT, further improved performance through advanced techniques, including dynamic masking mechanism and enhanced long-range dependency modeling [34].

The labeled data was then split into a training set of 12,000 texts and a testing set of 3000 texts. For the fine-tuning of RoBERTa, we used the AdamW optimizer with an L2 regularization coefficient of 0.2, cross-entropy loss, a learning rate of 1e-5 for 20 epochs and a batch size of 64. The 5-fold cross-validation experiment, detailed in Table S1, demonstrated that the classifier achieves accuracy and Macro-F1 of around 80%, suggesting sufficient competence in fine-grained emotion classification.

### 2.3. Theme Network Modeling

This study innovatively integrates theme analysis and word co-occurrence networks to construct a theme network, which not only clearly presents the distribution of vocabulary within each theme but also effectively highlights the correlations between various themes, thereby providing a more comprehensive analytical perspective. After completing data preprocessing, the latent topic structure was mined using the Latent Dirichlet Distribution (LDA) model [35]. LDA, as a probabilistic generative model based on Bayesian inference, has achieved robust performance in prior studies on Chinese datasets [36]. Its core assumption is that each document is composed of a mixture of potential topics, with each topic corresponding to a specific probability distribution over the vocabulary. Specifically, LDA generates a document-topic distribution matrix and a topic-term distribution matrix through statistical analysis of the document-term matrix. The probability of a corpus under the LDA model is mathematically defined as:

(1) $$p(W|\alpha ,\beta )=\;\prod_{d=1}^{M}\int p\left(\theta_{d}|\alpha \right)\left(\prod_{n=1}^{N_{d}}\sum_{z_{dn}}p\left(z_{dn}|\theta_{d}\right)p\left(w_{dn}|z_{dn},\beta \right)\right)d\theta_{d}$$

Here, *W* represents the corpus, $\theta_{d}$ is the topic distribution of document *d*, $z_{dn}$ is the topic assignment for the *n*-th word in the document, and $w_{dn}$ is the observed word. The Dirichlet prior parameters *α* and *β* are crucial for regulating the topic distributions and controlling the sparsity of document-topic and topic-term associations.

To determine the optimal number of topics (*K*), this study used an iterative method, gradually increasing *K* across multiple experiments while monitoring changes in perplexity and topic coherence scores [37,38]—key metrics for evaluating model-data fit. The experimental results showed that the majority of emotion-related topics displayed stably low perplexity and high coherence within the range of *K* = 3 to 6. Consequently, the number of topics was standardized to *K* = 4 to facilitate clearer comparisons across different groups. Additionally, the passes of the model was set to 20 to enhance its stability and ensure convergence and accuracy of the results. For each extracted topic, this study selected the top 15 keywords with the highest probabilities as representative terms. Based on this, a co-occurrence network was constructed where nodes represent topic tags and keywords, edges represent the membership relationships between topics and their keywords, and node size reflects the degree value (i.e., the number of connected edges). Finally, the Louvain algorithm using modularity maximization was applied to detect the community structures in the network, visualizing clusters via color-coding for enhanced interpretability. Notably, the LDA model was implemented using Python (version 3.6.3)’s Gensim library, while community detection was conducted using Gephi (version 0.9.2)’s built-in features.

### 2.4. Emotional Network Construction

Research has shown that communities on social media often form around well-known opinion leaders, who are typically popular individuals, celebrities, or organizations [39]. This concept stems from the two-step flow theory of communication [40], which suggests that new information is first transmitted through mass media to opinion leaders, and then spread to their respective social circles, ultimately influencing public perceptions. Although subsequent studies have questioned this theory and developed multi-step flow models [41], the rise of social media provides new channels for opinion leaders to exercise influence [42]. Bergström and Jervelycke Belfrage have confirmed that opinion leaders occupy a central position in the process of social media news dissemination [43], and users generally believe that they have a significant impact on information filtering. User influence essentially reflects their interactive abilities, so this study identifies influencers from the perspective of interactive networks. On Weibo, interactions such as following, mentioning and retweeting provide a foundation for influence analysis. Notably, retweeting behavior, while not always indicating agreement, shows that the retweeter pays attention to the message and acknowledges the informational value of retweetee [39]. Using retweeting relationship data, this study employs in-degree centrality—that is, counting the number of times each user is retweeted—to quantify user influence in emotional communication. This method is a common approach for identifying super-spreaders among information sources [39,44], aligning with the research goals of communication dynamics.

Specifically, after completing the fine-grained emotion classification of Weibo texts, this study constructs directed weighted retweeting networks for five emotions including anger and fear, denoted as *G* = (*V*, *E*), where the node set *V* = {*v*_1_, *v*_2_, …, *v_N_*} represents users and the edge set *E* = {*e*_1_, *e*_2_, …, *e_M_*} represents retweeting relationships, and the edge weights indicate the frequency of corresponding retweeting relationships. The size of the 10 constructed networks is detailed in Table S2. Then, a method based on in-degree centrality was used to evaluate and rank user importance, with all related calculations conducted using the Python NetworkX package. Finally, by gathering highly retweeted tweets of top 10 super-spreaders, this study further explores their impact on public emotions. The analysis of representative texts helps to reveal their specific role in emotional communication (See Tables S3–S17).

Here, we employ the concept of structural entropy from structural information theory to quantify the complexity of retweeting networks and the uncertainty of information propagation [45]. Structural entropy has been widely applied in graph analysis in recent years and has demonstrated excellent performance [46,47]. Similar to Shannon entropy, structural entropy primarily describes the uncertainty of random walks in complex networks. Its one-dimensional structural entropy is defined as follows:

(2) $$H\left(G\right)=-\underset{v\in G}{\sum }\frac{d\left(v\right)}{2M}{log}_{2}\frac{d\left(v\right)}{2M}$$

where *d*(*v*) represents the degree of node *v* in graph *G*. The two-dimensional structural entropy is derived by treating each community in a network as a point, extending the concept of one-dimensional structural entropy. It is partitioned based on network communities and serves to describe the stability of the entire network after partitioning. Specifically, the network can be divided into *P* = (*X*_1_, *X*_2_, …, *X_n_*), where for any vertex *v* in graph *G*, there exists exactly one *j* ∈ [1, *L*], such that *v* ∈ *X_j_*. Thus, the two-dimensional structural entropy is defined as:

(3) $$\begin{array}{ll} H^{P}\left(G\right)= & \overset{L}{\underset{j=1}{\sum }}\frac{V_{j}}{2M}H\left(\frac{d_{i}^{\left(j\right)}}{V_{j}},\ldots ,\frac{d_{n_{j}}^{\left(j\right)}}{V_{j}}\right){-\overset{L}{\underset{j=1}{\sum }}\frac{g_{j}}{2M}log}_{2}\frac{V_{j}}{2M}\\ & \;\;=-\overset{L}{\underset{j=1}{\sum }}\frac{V_{j}}{2M}\overset{n_{j}}{\underset{i=1}{\sum }}\frac{d_{i}^{\left(j\right)}}{V_{j}}{log}_{2}\frac{d_{i}^{\left(j\right)}}{V_{j}}{-\overset{L}{\underset{j=1}{\sum }}\frac{g_{j}}{2M}log}_{2}\frac{V_{j}}{2M} \end{array}$$

where *V_j_* represents the “volume” of community *X_j_*, which is the sum of the degrees of all nodes in *X_j_*. *g_j_* represents the degree of each community *X_j_*, which is the number of edges connecting nodes within *X_j_* to other communities. *n_j_* is the number of nodes in *X_j_*, and $d_{i}^{\left(j\right)}$ is the degree of the *i*-th node in community *X_j_*. While one-dimensional structural entropy is based solely on the degree distribution of all nodes in the network, two-dimensional structural entropy measures both the uncertainty of connections between communities and within communities, making it more suitable for evaluating the stability of real network communities. Lower entropy in the two-dimensional structure of the network indicates a clearer community structure and simpler connection paths between nodes. Conversely, higher entropy suggests a more complex and diverse network structure, with greater efficiency in cross-community propagation. Therefore, we integrate the Louvain algorithm with structural entropy to comprehensively evaluate the structure of retweeting networks. Specifically, we employed the Louvain method from Python’s Community package, which partitions the graph into weighted communities based on the sum of weighted degrees.

## 3. Results

### 3.1. Overall Emotional Comparison

**Figure 1:**
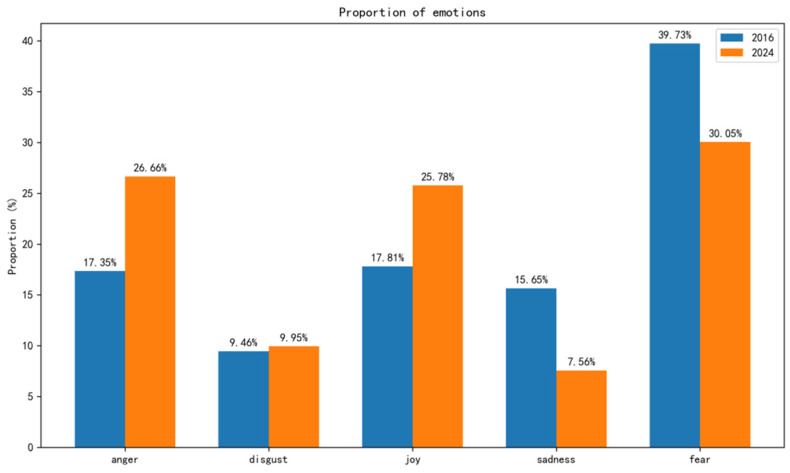
Comparison of emotional proportions between 2016 and 2024.

**Figure 2:**
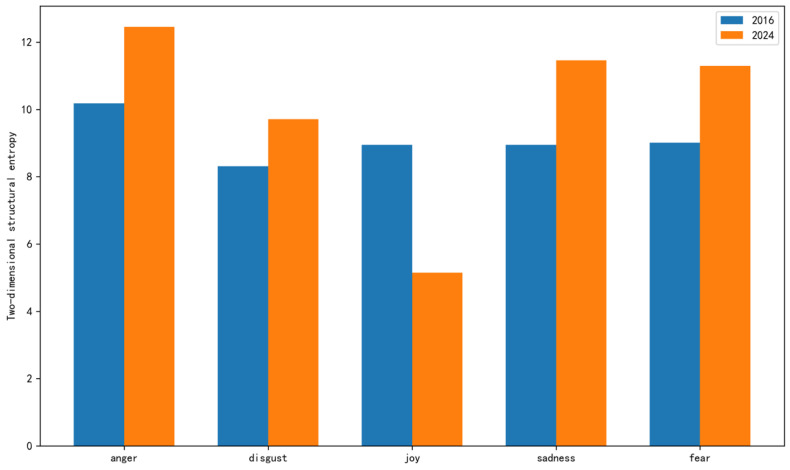
Comparison of structural entropy of emotional communication between 2016 and 2024.

This study employed a deep learning model to conduct fine-grained emotion recognition on collected HPV-related tweets. Regarding RQ1, Figure 1 illustrates the changes in the distribution of fine-grained emotions in tweets over the years. Although the overall proportion of various negative emotions was relatively high in both 2016 and 2024, the proportion of positive emotion in comments about HPV vaccines increased in 2024. In contrast, the proportion of some discrete negative emotions, such as sadness and fear, decreased, likely due to the public’s enhanced understanding of HPV from promotional activities, which alleviated excessive concerns and panic. It is worth noting that the proportion of anger showed a notable increase in 2024, suggesting a potential shift in discussion topics. As illustrated in Figure 2, there is a notable difference in the two-dimensional structural entropy values of retweeting networks between 2016 and 2024. In 2016, the entropy values across various emotions were relatively uniform. However, by 2024, while the entropy for joy significantly decreased, that for all other emotions increased. This transformation suggests that the propagation of emotions, excluding joy, has become more complex and diverse by 2024.

### 3.2. Semantic Network Analysis of Emotional Themes

**Figure 3:**
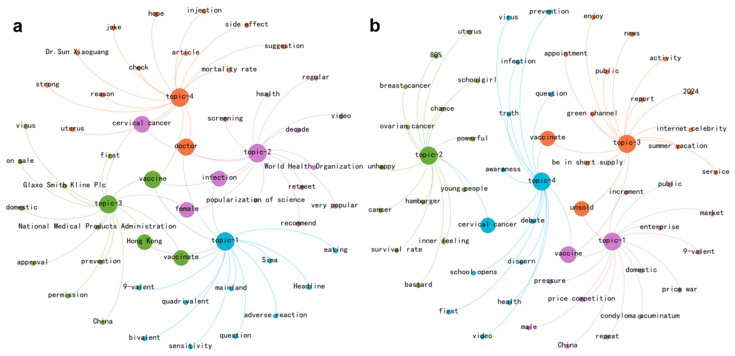
Thematic network visualization of anger discussions. Note that sub-graphs (**a**,**b**) represent 2016 and 2024, respectively.

In addressing RQ2, this study compared the central themes of various emotions and the associations between theme words in 2024 and 2016 using LDA topic modeling and word co-occurrence networks. Figure 3 shows that in 2016, anger-related keywords primarily include “vaccinate”, “cervical cancer” and “on sale”, reflecting public interest in the sale of HPV vaccines and the demand for popularization of vaccine effects at that time. By 2024, the main keywords had expanded to encompass “unsold”, “male” and “price competition”. Based on Table S13, these keywords can be traced back to relevant popular comments, such as “companies targeting the male market” from Sina News and “plan to deceive men into getting vaccinated” from individual bloggers, reflecting widespread dissatisfaction with the recent shift in the HPV vaccine industry towards male marketing. These comments further raise concerns that this strategy may be driven by profit motives, aimed at expanding the vaccination population to reduce excess inventory.

**Figure 4:**
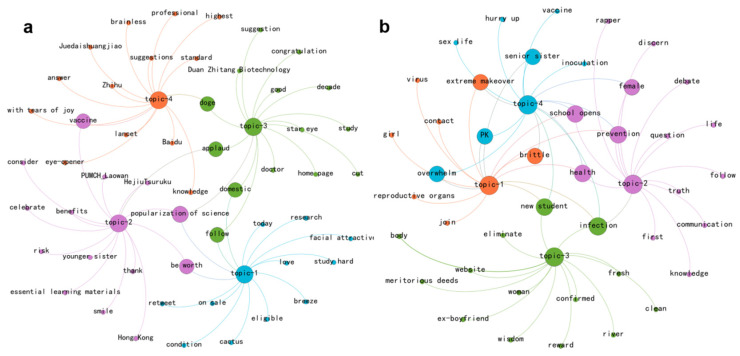
Thematic network visualization of joy discussions. Note that sub-graphs (**a**,**b**) represent 2016 and 2024, respectively.

As depicted in Figure 4, the predominant themes associated with joy in 2016 revolved around science popularization, whereas in 2024, a variety of new topics surfaced, including campus activities and artistic expressions, which infused the discourse with vitality and positive sentiment. Key terms like “extreme makeover”, “senior sister”, “new student”, “school opens”, “brittle” and “PK” suggest that campus initiatives employed gamified and youthful terminology (e.g., “Brittle college girls should Strengthen Herself” and “#HPV Prevention Health Extreme Makeover PK Competition#”) to encourage collective engagement in campus health initiatives (see Table S14), thereby fostering mobilization and a sense of community. Addressing the stigma surrounding women with HPV, female rapper Yu Zhen (ingrita.yuzhen) lightheartedly quipped on a talk show, “I am honored to spread some knowledge about HPV vaccines to everyone, but I think some male rappers are the true ambassadors for HPV transmission”, employing a casual and distinctive style to redirect the blame for HPV infection from individual women to broader social transmission dynamics, and using entertaining narratives to dispel traditional stigmatized perceptions, such as “vaccination is necessary only when private life is chaotic”.

Public fear mainly arises from HPV-related cancers and misconceptions regarding vaccine side effects. Notably, the significant increase in the frequency of keywords such as “male” and “schoolboy” in 2024 suggests that public discourse on male HPV vaccination has partially alleviated the fear issues previously concentrated on the female population. According to Figure S4, the recurrent appearance of keywords like “examination” and “treatment” across multiple themes in 2024 indicates a shift in public attention towards medical prevention and treatment, rather than solely on the anxiety associated with infection consequences. While the proportion of disgust in HPV discussions remained relatively stable between 2016 and 2024 (see Figure S5), there were significant differences in their thematic connotations and contexts. The discussions in 2024 are richer in content, as demonstrated by the clustering of keywords such as “physical examination”, “gynecology” and “doctor”, which reflect that negative experiences related to gynecological examinations and vaccination processes have become new triggers for disgust. Notably, the keywords associated with various emotions in 2016 uniformly included “popularization of science”, underscoring the public’s pressing need for relevant knowledge in the early stage of vaccine launch. This also reflected the concentrated nature of topics during that period, where identical information sources could provoke varied emotional responses among the public. In contrast, discussions in 2024 exhibited a more significant diversification of topics.

### 3.3. Network Analysis of Emotional Communication

**Figure 5:**
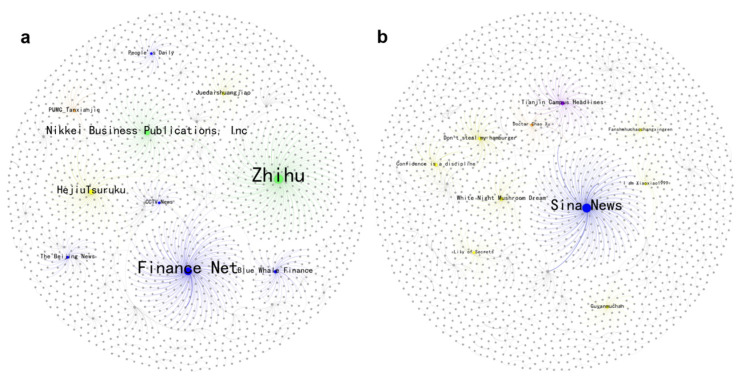
The network of anger spreading. Note that sub-graphs (**a**,**b**) represent 2016 and 2024, respectively. The size of each node reflects its number of retweeters, and the top 10 super-spreaders are colored based on their verified type: blue for media, green for enterprises, orange for celebrities, purple for campuses and yellow for influential bloggers. The color of each edge matches that of its source node.

RQ3 examines the differences in the emotional impact of various super-spreaders on retweeters. Based on this, Figure 5 compares retweeting networks for anger discussions on Weibo between 2016 and 2024, while Tables S3 and S4 provide a detailed ranking of super-spreader influence based on repost counts. In the retweeting network of 2016, many media and enterprise accounts were core nodes, playing an important role in disseminating information about the approval of HPV vaccine for sale (see Table S13). In 2024, in addition to traditional media such as Sina News continuing to play an important role, the emergence of a large number of individual bloggers has become a key feature. These bloggers typically focus on specific fields or themes, with their own fan base and a certain level of influence. For example, bloggers such as “White Night Mushroom Dream” question whether the male market offers new growth potential for HPV vaccines or express concerns over pricing and promotions, as seen in highly retweeted comments like “HPV is mainly due to its exorbitant price” and “plan to deceive men into getting vaccinated”, which drive the spread of public anger. These finely categorized individual bloggers have further fueled the spread of anger by utilizing richer themes such as gender-differentiated vaccination strategies and their own choices. Their comments, often personalized and critical, tend to resonate with targeted audiences.

**Figure 6:**
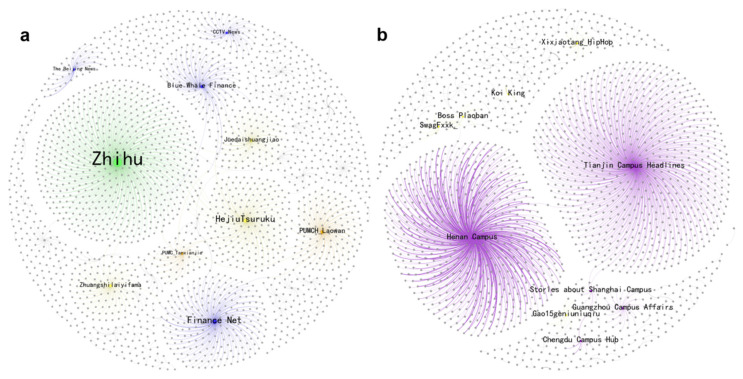
The network of joy spreading. Note that sub-graphs (**a**,**b**) represent 2016 and 2024, respectively. The size of each node reflects its number of retweeters, and the top 10 super-spreaders are colored based on their verified type: blue for media, green for enterprises, orange for celebrities, purple for campuses and yellow for influential bloggers. The color of each edge matches that of its source node.

Figure 6 presents the joy retweeting network. Notably, Tables S3 and S5 show a 90% overlap between the top 10 super-spreaders of joy and those of anger in 2016. These influential accounts were mostly media or medical experts, such as Finance Net and PUMC_Tanxianjie, a professor at Peking Union Medical College. This indicates that the observed emotional disparities at that time were not due to the differentiation of information sources but rather the endogenous transformation of the public based on their pre-existing knowledge and stances after receiving information. For example, the announcement of the HPV vaccine’s approval could trigger both expectations for medical advancements (joy) and skepticism about the timeliness of the approval process (anger), reflecting early public cognitive conflicts regarding the HPV-related issue. By 2024, the landscape had shifted, with campus accounts, such as Henan Campus and Tianjin Campus Headlines, and music bloggers like Xixiaotang-HipHop, leading the dissemination of public joy. This trend is consistent with the results of our thematic analysis, underscoring the pivotal role of campus-led initiatives in enhancing public awareness of the HPV vaccine.

As illustrated in Figures S6 and S7, the spread of fear and sadness in 2024 is primarily driven by Dr. Chao Xu and individual bloggers. Interestingly, these super-spreaders often share followers-submitted cases to spark public discussion and emotional expression. Some images and descriptions can directly cause fear, while certain experiences can evoke sadness and sympathy. Figure S8 shows that disgust in 2024 was mainly triggered by uninspiring surveys from enterprise and medical examination experiences shared by blogger Ms. Laoyao, aligning with thematic analysis findings. Notably, HPV infection cases shared by submitter often trigger public discussions on health responsibility, which can lead to gender opposition and vaccine stigmatization. For example, in the comments of health bloggers such as Yutouweibo, the phrases “practice sexual discipline” and “Clean women don’t need HPV vaccines” implicitly link HPV infection with morality. Although terms like “ganjing (cleanliness)”, “ziai (self-care)”, and “promiscuity”, noted by Li et al. for reflecting Chinese gender norms [48], appear in online HPV discussions, these biased terms have low frequency and are not identified as core themes by topic models, indicating that HPV vaccine stigmatization remains limited on Weibo.

## 4. Discussion

During the critical period of the Healthy China 2030 strategy and the widespread adoption of HPV vaccines [49,50], social media has emerged as a key platform influencing public cognition, emotions, and vaccination decisions, and its underlying dynamics urgently require further exploration. This study used techniques such as fine-grained emotion recognition, topic network analysis, and retweeting network tracking to systematically analyze the public emotion structure, topic evolution, and transmission paths of nearly 40,000 tweets from 2016 and 2024. For the first time, this study comprehensively delineated the dynamic evolution of the Chinese public’s understanding of and fine-grained emotions toward HPV vaccines. The results found that the distribution of public emotions had significantly changed over time. Specifically, public anger over commercialization concerns and gender imbalance in vaccination saw a significant increase, while positive emotion significantly increased in contexts such as university promotional activities. The evolution of structural entropy across different emotions indicates that the joy propagation network exhibits a more concentrated structure, with information primarily circulating within a few communities, thereby forming an echo chamber effect. In contrast, the increased entropy for other emotions reflects a diversification of communication modes, enhancing their efficiency in disseminating information across communities, which promotes broader spread of information. Notably, various emotional communication networks and thematic distributions had undergone structural transformation. Communication has shifted from being dominated by traditional media and experts to being driven by segmented bloggers, who have transitioned discussion from relatively unified themes such as science popularization and HPV-related diseases to diversified issues, thereby amplifying the spread of various emotions [51]. This study uncovers the synergistic evolution patterns of the emotion-theme-communicator triad and develops a dynamic analysis framework for public health communication research in the digital age. These findings have important theoretical and practical implications for evidence-based optimization of public health policies.

From 2016 to 2024, public anger concerning the HPV vaccine on Chinese social media underwent significant changes. Initially, when media outlets primarily disseminated vaccine marketing information, the public retained a certain level of trust in relevant institutions, and anger primarily stemmed from individual cognitive conflicts, such as skepticism about approval processes and divergence between expected and actual vaccine outcomes. By 2024, this anger had pivoted to distrust in vaccine commercialization. The transformation in keywords—from terms like “vaccinate” and “on sale” to “unsold”, “price competition” and “deceive men into getting vaccinated”—along with public discourse, clearly indicated that the industry’s expansion into the male market was widely perceived as a profit-driven strategy by pharmaceutical companies, aimed at clearing excess inventory rather than addressing public health needs or gender equity, thereby precipitating a “commercial trust crisis”. Zhou et al. pointed out that the promotion of male HPV vaccine policies in China was slow [52], compounded by insufficient health education. Our findings highlight the coexistence of male vaccine policy promotion and the shortage of 9-valent vaccines for females, which led the public to view male-targeted promotion as “inventory clearance” rather than equitable distribution. This perception exacerbated the “uncertainty” emphasized by innovation diffusion theory and shifted public attention from vaccine efficacy to promotional motives [53]. Concurrently, the dissemination of anger underwent a key transformation: communication shifted from media dominance to being driven by numerous influential bloggers who engaged specific audiences through personalized, critical content focused on male-targeted strategies and commercial incentives. Such content was highly likely to spread and reinforce skeptical narratives within closed and homogeneous online communities [21], significantly amplifying anger. Moreover, public inquiries into commercialization motives were not responded timely or effectively, leading to the accumulation of negative emotions within communities. In sum, this study confirms that Chinese public’s anger primarily stems from dissatisfaction with vaccine commercialization and pricing, which is different from U.S. vaccination hesitancy driven by the “feminization” of HPV vaccines [54]. Additionally, anger was exacerbated by domain-segmented bloggers who amplified dissemination in closed networks, revealing a new risk of public trust erosion towards official media in health communication. These findings provide insights for future health communication strategies focused on “trust repair”—through enhanced transparency, timely responses to concerns, and balancing commercial interests with public welfare—while advocating for more interactive communication models.

In 2024, the themes of positive emotion (joy) related to the HPV vaccine on Weibo had significantly diversified, extending beyond the predominant focus on science popularization observed in 2016. This evolution underscores the effectiveness of innovative communication models in health education. On the one hand, university campuses emerged as pivotal settings, where HPV prevention knowledge was integrated into “collective action symbols” through gamified and youth-oriented health initiatives such as “extreme makeover”, “brittle”, and “PK Competition” [55]. These initiatives not only effectively increase the attention of college students, but also internalize vaccine acceptance as a youth identity, thereby addressing the limitations of traditional, one-way science popularization efforts. On the other hand, artistic expression played a significant role in destigmatization efforts, exemplified by rapper Yu Zhen’s humorous storytelling on talk shows. Specifically, Yu Zhen’s jest about “male rappers as HPV transmission ambassadors” cleverly utilized the “responsibility allocation” framework from risk culture theory [56], shifting the responsibility for infection from individual women to broader societal factors. This approach not only deconstructed the “feminization” of HPV vaccines but also mitigated associated gender biases through a lighthearted cultural narrative [48,57], marking a significant step towards destigmatization. These developments highlight the growing influence of non-traditional communicators, such as campus accounts and music bloggers, who leverage personalized and engaging content to address sensitive health topics. By doing so, they not only enhanced public engagement and participation but also demonstrated the unique advantages of such communicators in navigating complex issues, effectively compensating for the shortcomings of official institutions in interactive storytelling [58]. Ultimately, this shift in communication strategies has significantly expanded the diversity and effectiveness of health communication efforts.

Additionally, the widespread discussion on male HPV vaccination has partially alleviated the fear and pressure previously concentrated on the female population [48], while discussions shifted towards medical prevention behaviors such as “examination” and “treatment”, reflecting a more pragmatic public risk perception. Specifically, emotions of sadness and fear were mainly driven by opinion leaders such as Dr. Chao Xu, through sharing user cases and stories, aligning with the storytelling mechanism in health communication that triggers emotional resonance. Despite the broader dissemination of such content, it risks fostering gender bias, as seen in comments like “clean women do not need vaccines” and “practice sexual discipline”, moralistic disease-related rhetoric, and subtle stigma toward vaccine recipients. Such remarks implying “promiscuity” and “self-care” may exacerbate women’s psychological burden and suppress men’s willingness to get vaccinated, highlighting the double-edged sword effect of narrative dissemination. Concurrently, triggers of disgust had shifted from abstract disease cognition to negative experiences in specific medical scenarios. Cases like the physical examination disclosed by blogger Ms. Laoyao drove public disgust towards the chaos of medical commercialization rather than the vaccines themselves, confirming the amplification effect of offline service defects on online emotions [21]. Notably, this study confirms that current male issues have moved from marginalization to the center of controversy, consistent with Zhou et al. [52]. Previous studies have criticized the lack of awareness and scientific education regarding male vaccination, but this study reveals that the dominant issue related to men, “excessive commercialization”, conflated gender equality demands with corporate commercialization motives, potentially hindering rational discussion. Thus, there is an urgent need to address the shortcomings of official systems through innovative forms, such as transparency and interactivity, to rebuild public trust.

Based on the findings, this study proposes a comprehensive set of strategies to optimize HPV vaccine promotion and guide public opinion effectively. First, the government should establish stricter vaccine approval and regulatory mechanisms to ensure transparency in vaccine pricing and policies, thereby preventing enterprises from exploiting information asymmetry for undue profits. Simultaneously, the government can promptly understand public concerns about commercialization and gender equality issues by real-time monitoring public opinion on social media, enabling timely interventions such as enhancing support for the male HPV vaccine market and health education to prevent misconceptions like “clearance promotions”. Second, responsible enterprises should strengthen their management, transparently provide vaccine pricing standards and their rationale, and disclose relevant policies and processes to avoid public distrust due to lack of information. To rebuild trust, they must address public concerns swiftly and improve service quality by adopting technological solutions like online appointment and vaccination tracking to minimize disputes during vaccination. Third, mainstream media should shift from traditional one-way communication to interactive engagement with the public. On the one hand, it is essential to develop innovative storytelling methods that are accessible, vivid, and engaging to effectively disseminate knowledge about the HPV vaccine and enhance its acceptance by the public. On the other hand, the media should actively fulfill their supervisory role by investigating and reporting on potential issues to prompt relevant enterprises to address them, and by promoting expert discussions to foster informed and rational public dialog. Fourth, influential bloggers should enhance their knowledge and scientific understanding of the HPV vaccine, sharing accurate and unbiased information to correct public misconceptions and foster positive vaccination attitudes. Furthermore, the public should actively engage in overseeing policy implementation, offering constructive feedback to improve transparency and accountability within the sector. Collaboration among government, enterprises, media, and citizens is vital to promote the dissemination of accurate HPV vaccine information, safeguard public health, and maintain social stability.

This study inevitably has several limitations. First, data sourced from Weibo may not fully cover the entire population, particularly as demographics with lower social media engagement, such as the elderly, are underrepresented, potentially skewing findings towards the perspectives of younger individuals. Second, the absence of detailed user demographics, including age and income, impedes the ability to compare attitudes across diverse groups. Third, this study is mainly based on users’ online public comments and emotional reactions, making it difficult to gain a deep understanding of their inner thoughts and complex perspectives. Future promising work should collect in-depth opinions and detailed personal information from comprehensive population surveys to overcome the above limitations. Moreover, with the ongoing advancement of artificial intelligence technology, future studies can leverage these advanced tools to improve the accuracy and efficiency of emotion analysis and combine experimental interventions such as testing the relief effect of different narratives on negative emotions to further optimize health communication strategies.

## 5. Conclusions

In summary, this study systematically examines the evolution of emotional structures, themes, and key communicators in public discourse on HPV vaccination from 2016 to 2024. The core findings indicate that public anger has shifted from early individual doubts to systematic questioning of vaccine commercialization motives, thereby exposing new risks of “trust rupture”. Meanwhile, the emotional communication network displays polarized characteristics: joy tends to create an echo chamber with concentrated information flow, whereas other emotions show higher cross-community communication efficiency due to their fragmented communication structures. Additionally, positive emotions demonstrate diverse expressions in university promotional activities and destigmatization narratives. The research confirmed that the communication ecosystem transitioned from being dominated by traditional media to being driven by segmented bloggers, which significantly amplified the intensity of emotional communication and promoted the fragmentation of themes. These findings not only address the gap in comprehensively understanding the dynamics of HPV vaccine discussions on social media in China, but also uncover the co-evolutionary patterns of the emotion-theme-communicator triad, which underscores the urgent need to repair public trust and innovate communication strategies in the era of focus communication. This study provides a theoretical framework and practical insights for optimizing public health communication, guiding rational public opinion, and promoting vaccine equity and accessibility.

## Data Availability

The datasets used in this study are publicly available and can be downloaded freely through https://github.com/bowenshi/hpv-emotion-analysis (accessed on 3 June 2025).

## Supplementary Materials

The following supporting information can be downloaded at: https://www.mdpi.com/article/10.3390/e27090887/s1, Figure S1: Number of HPV-related tweets per day in 2016; Figure S2: Number of HPV-related tweets per day in 2024; Figure S3: Thematic network visualization of fear discussions; Figure S4: Thematic network visualization of sadness discussions; Figure S5: Thematic network visualization of disgust discussions; Figure S6: The network of fear spreading; Figure S7: The network of sadness spreading; Figure S8: The network of disgust spreading; Table S1: The precision, recall and F-measure of the cross-validation; Table S2: Size statistics of each retweeting network; Table S3: Top 10 super-spreaders in the anger spreading network of 2016; Table S4: Top 10 super-spreaders in the anger spreading network of 2024; Table S5: Top 10 super-spreaders in the joy spreading network of 2016; Table S6: Top 10 super-spreaders in the joy spreading network of 2024; Table S7: Top 10 super-spreaders in the fear spreading network of 2016; Table S8: Top 10 super-spreaders in the fear spreading network of 2024; Table S9: Top 10 super-spreaders in the sadness spreading network of 2016; Table S10: Top 10 super-spreaders in the sadness spreading network of 2024; Table S11: Top 10 super-spreaders in the disgust spreading network of 2016; Table S12: Top 10 super-spreaders in the disgust spreading network of 2024; Table S13: Representative examples of popular tweets from super-spreaders in anger spreading network; Table S14: Representative examples of popular tweets from super-spreaders in joy spreading networks; Table S15: Representative examples of popular tweets from super-spreaders in fear spreading networks; Table S16: Representative examples of popular tweets from super-spreaders in sadness spreading networks; Table S17: Representative examples of popular tweets from super-spreaders in disgust spreading networks.
