# Supplementary material for: Evolving Public Attitudes Towards the HPV Vaccine in China: A Fine-Grained Emotion Analysis of Sina Weibo (2016 vs. 2024)
Source: Entropy (Basel). 2025 Aug 22;27(9):887. doi: 10.3390/e27090887 (PMC12469185; doi:10.3390/e27090887)
Supplement: Supplementary file 1 [file entropy-27-00887-s001.zip › entropy-3772309-supplementary.pdf]

Supplementary Information for:
Evolving Public Attitudes Towards the HPV Vaccine in China: A Fine-Grained Emotion Analysis of Sina Weibo (2016 vs. 2024)

This document includes:

Supplementary Tables S1-S17,
Supplementary Figures S1-S8.

Supplementary Table S1. The precision, recall and F-measure of the cross-validation. All refers to considering all emotions as a whole.

Emotion	Precision (%)	Recall (%)	F-measure (%)
Anger	72.42	81.83	76.84
Disgust	74.03	67.00	70.34
Joy	85.08	77.00	80.84
Sadness	82.85	81.33	82.09
Fear	86.36	92.83	89.48
All	80.15	80.00	79.92

Supplementary Table S2. Size statistics of each retweeting network.

Emotion	2016		2024	
	Nodes	Edges	Nodes	Edges
Anger	1,784	1,668	1,843	1,573
Disgust	1,142	1,032	928	776
Joy	2,216	2,078	1,765	1,621
Sadness	2,001	1,871	647	504
Fear	4,210	4,191	2,133	1,919

Supplementary Table S3. Top 10 super-spreaders in the anger spreading network of 2016.

Rank	User name	Verified type	User description
1	Zhihu	enterprise	A Quora-type question and answer site
2	Finance Net	media	website, http://www.caijing.com.cn/
3	Nikkei Business Publications, Inc	enterprise	A book and magazine publisher based in Tokyo, Japan
4	HejiuTsuruku	ordinary	Science popularization blogger
5	Blue Whale Finance	media	A platform for original financial news reports and financial information services based in Shanghai
6	Juedaishuangjiao	ordinary	Health blogger
7	The Beijing News	media	Chinese Communist Party newspaper
8	PUMC_Tanxianjie	celebrity	Professor of Peking Union Medical College (PUMC)
9	CCTV News	media	China Central Television
10	People’s Daily	media	Official newspaper of the Chinese Communist Party

Supplementary Table S4. Top 10 super-spreaders in the anger spreading network of 2024.

Rank	User name	Verified type	User description
1	Sina News	media	The official account of Sina Weibo, providing domestic and international news and information
2	Don’t steal my hamburger	ordinary	Video blogger
3	White Night Mushroom Dream	ordinary	Video blogger
4	Tianjin Campus Headlines	Campus	Campus Information in Tianjin
5	Confidence is a discipline	ordinary	Video blogger
6	Guyanmuchan	ordinary	Technology blogger
7	Doctor Chao Xu	celebrity	Attending physician of the Department of Urology at Reproductive Hospital Affiliated to Shandong University
8	Fanshehuchaochangxingren	ordinary	Anime blogger
9	Lily of Secrets	ordinary	Emotional blogger
10	I am Xiaoxiao1999	ordinary	Video blogger

Supplementary Table S5. Top 10 super-spreaders in the joy spreading network of 2016.

Rank	User name	Verified type	User description
1	Zhihu	enterprise	A Quora-type question and answer site
2	Finance Net	media	website, http://www.caijing.com.cn/
3	HejiuTsuruku	ordinary	Science popularization blogger
4	PUMCH_Laowan	celebrity	Chief physician of the Obstetrics and Gynecology Department at Peking Union Medical College Hospital (PUMCH)
5	Blue Whale Finance	media	A platform for original financial news reports and financial information services based in Shanghai
6	Juedaishuangjiao	ordinary	Health blogger
7	Zhuangshilaiyifama	ordinary	Humor blogger
8	CCTV News	media	China Central Television
9	PUMC_Tanxianjie	celebrity	Professor of Peking Union Medical College (PUMC)
10	The Beijing News	media	Chinese Communist Party newspaper

Supplementary Table S6. Top 10 super-spreaders in the joy spreading network of 2024.

Rank	User name	Verified type	User description
1	Henan Campus	Campus	Campus Information in Henan
2	Tianjin Campus Headlines	Campus	Campus Information in Tianjin
3	Guangzhou Campus Affairs	Campus	Campus Information in Guangzhou
4	Chengdu Campus Hub	Campus	Campus Information in Chengdu
5	Xixiaotang_HipHop	ordinary	Music blogger
6	Stories about Shanghai Campus	Campus	Campus Information in Shanghai
7	SwagFxxk_	ordinary	Music blogger
8	Gao15geniuniuqiu	ordinary	Makeup blogger
9	Boss Plaoban	ordinary	Music blogger
10	Koi King	ordinary	Video blogger

Supplementary Table S7. Top 10 super-spreaders in the fear spreading network of 2016.

Rank	User name	Verified type	User description
1	Finance Net	media	website, http://www.caijing.com.cn/
2	Zhihu	enterprise	A Quora-type question and answer site
3	Juedaishuangjiao	ordinary	Health blogger
4	Guokr Net	enterprise	A pan technology interest community website
5	HejiuTsuruku	ordinary	Science popularization blogger
6	People’s Daily	media	Official newspaper of the Chinese Communist Party
7	CCTV News	media	China Central Television
8	PUMC_Tanxianjie	celebrity	Professor of Peking Union Medical College (PUMC)
9	Nikkei Business Publications, Inc	enterprise	A book and magazine publisher based in Tokyo, Japan
10	Chengdu Business Daily	media	A newspaper based in Chengdu, Sichuan Province, China

Supplementary Table S8. Top 10 super-spreaders in the fear spreading network of 2024.

Rank	User name	Verified type	User description
1	Will fire	ordinary	Entertainment Blogger
2	Qiao Hongyu	ordinary	Makeup blogger
3	Yutouweibo	ordinary	Health blogger
4	Sichuan Prison	government	Official Weibo account of Sichuan Provincial Prison Administration Bureau
5	Zoe's mother from Germany	ordinary	Health blogger
6	Apufferfishcanalwayscope	ordinary	Sports blogger
7	Zhang Yu from Medical Science Popularization	celebrity	Oncology physician at Peking University Third Hospital
8	Sina Hotspots	media	Sina official platform releases hot topics in real-time
9	Doctor Chao Xu	celebrity	Attending physician of the Department of Urology at Reproductive Hospital Affiliated to Shandong University
10	Ice Snake Emperor	ordinary	Reading blogger

Supplementary Table S9. Top 10 super-spreaders in the sadness spreading network of 2016.

Rank	User name	Verified type	User description
1	Zhihu	enterprise	A Quora-type question and answer site
2	Finance Net	media	website, http://www.caijing.com.cn/
3	Nikkei Business Publications, Inc	enterprise	A book and magazine publisher based in Tokyo, Japan
4	CCTV News	media	China Central Television
5	HejiuTsuruku	ordinary	Science popularization blogger
6	Zhuangshilaiyifama	ordinary	Humor blogger
7	Guokr Net	enterprise	A pan technology interest community website
8	The Beijing News	media	Chinese Communist Party newspaper
9	Sanlian Lifeweek	media	News magazine based in Beijing, China, known for its in-depth reporting and cultural commentary
10	Juedaishuangjiao	ordinary	Health blogger

Supplementary Table S10. Top 10 super-spreaders in the sadness spreading network of 2024.

Rank	User name	Verified type	User description
1	Doctor Chao Xu	celebrity	Attending physician of the Department of Urology at Reproductive Hospital Affiliated to Shandong University
2	Yezhini	ordinary	Emotional blogger
3	Ercha is wealthy and willful	ordinary	Food blogger
4	I think so. How about you	ordinary	Video blogger
5	Xiaoananyiyi	ordinary	Food blogger
6	Lotus seeds and white cranes	ordinary	Health blogger
7	Talking about reasoning freely	ordinary	Constellation blogger
8	Lieli	ordinary	Entertainment Blogger
9	Dominant Sister	ordinary	Emotional blogger
10	Fubao	ordinary	Emotional blogger

Supplementary Table S11. Top 10 super-spreaders in the disgust spreading network of 2016.

Rank	User name	Verified type	User description
1	Zhihu	enterprise	A Quora-type question and answer site
2	Finance Net	media	website, http://www.caijing.com.cn/
3	HejiuTsuruku	ordinary	Science popularization blogger
4	Juedaishuangjiao	ordinary	Health blogger
5	The Beijing News	media	Chinese Communist Party newspaper
6	CCTV News	media	China Central Television
7	PUMC_Tanxianjie	celebrity	Professor of Peking Union Medical College (PUMC)
8	Guokr Net	enterprise	A pan technology interest community website
9	PUMCH_Laowan	celebrity	Chief physician of the Obstetrics and Gynecology Department at Peking Union Medical College Hospital (PUMCH)
10	People’s Daily	media	Official newspaper of the Chinese Communist Party

Supplementary Table S12. Top 10 super-spreaders in the disgust spreading network of 2024.

Rank	User name	Verified type	User description
1	Fenbi Technology Ltd	enterprise	An online education and training enterprise based on mobile Internet
2	Ms. Laoyao	ordinary	Emotional blogger
3	Sichuan Prison	government	Official Weibo account of Sichuan Provincial Prison Administration Bureau
4	Zoe's mother from Germany	ordinary	Health blogger
5	Doctor Chao Xu	celebrity	Attending physician of the Department of Urology at Reproductive Hospital Affiliated to Shandong University
6	Galaxy Ferry	ordinary	Entertainment Blogger
7	I think so. How about you	ordinary	Story blogger
8	Zhangzuofeng_UCLA	celebrity	Professor of Epidemiology at University of California-Los Angeles
9	Xixiaotang_HipHop	ordinary	Music blogger
10	Mr. Six Floors	ordinary	Health blogger

Supplementary Table S13. Representative examples of popular tweets from super-spreaders in anger spreading network.

Time	User	Tweet
2024	Sina News	#九价 HPV 疫苗或将重演价格战#HPV 疫苗卖不动了，企业盯上男性市场#男性市场能否成 HPV 疫苗新的增量#据中国新闻周刊，国产 HPV 疫苗市场近期再度成为公众瞩目的焦点，价格战的硝烟弥漫，使得市场格局发生了深刻变化，随着多家上市疫苗企业公布上半年财报，业绩的大幅下滑揭示行业正面临的严峻挑战，面对价格竞争压力…
		#9-valent HPV vaccine may repeat price war# HPV vaccine is unsold, companies are targeting the male market # Can the male market become a new increment of HPV vaccine # According to China Newsweek, the domestic HPV vaccine market has once again become the focus of public attention recently, and the intense price war has caused profound changes in the market pattern. With multiple listed vaccine companies announcing their first half financial reports, the significant decline in performance reveals the severe challenges that the industry is facing. Faced with price competition pressure…
	White Night Mushroom Dream	HPV 主要是价格贵的离谱，疫苗接种绿色通道让她们无需预约即可享受接种服务，女生骗不动了，准备骗男生打了，反正我没打，洁身自好就不需要打，#HPV 疫苗卖不动了#
		HPV is mainly due to its exorbitant price. The green channel for vaccination allows women to enjoy vaccination services without the need for an appointment. Schoolgirls can’t be fooled anymore, so they plan to deceive men into getting vaccinated. Anyway, I didn’t get vaccinated, so I don't need to get vaccinated if I’m clean and self-disciplined. # HPV vaccine is unsold #
	Guyanmuchan	#HPV 疫苗卖不动了#我只说我自己没打，我也不会给我姑娘打，别的不说，怕被喷，资本很恐怖[允悲][允悲][允悲] 洁身自好（包括男女），每年体检比啥都重要，绿色通道让她们无需预约即可享受接种服务，界面新闻梳理公开报道发现，自 2024 年上半年，预约活动预计将覆盖超 3200 个接种点门诊。
		#The HPV vaccine is unsold# I just said that I haven’t get vaccinated, and I won't get my daughter vaccinated either. Besides, I’m afraid of backlash and capital is terrifying. [facepalm] [facepalm] [facepalm] Being clean and self-disciplined (including men and women), and getting annual physical examinations are more important than anything else. The green channel allows them to enjoy vaccination services without the need for appointments. According to public reports, from the first half of 2024, appointment activities are expected to cover more than 3200 vaccination clinics.
2016	Juedaishuangjiao	中国 CFDA 终于批准了 GSK 生产的 HPV 疫苗（俗称宫颈癌疫苗）、我看到个别主要媒体账号下竟然一片欢呼、大有谢煮隆恩的味道。人贱至此、我特么真的受不了了。我不爆粗口没法活了——谢什么？Shenny Marlugbee 啊谢？！多少年了？十年过去了！日本鬼子都从卢沟桥解甲归田去种越光大米了！相关机构你把中国人... http://m.weibo.cn/client/version
		China’s CFDA has finally approved the HPV vaccine (commonly known as cervical cancer vaccine) produced by GSK. I saw cheers and gratitude from some major media accounts. I can’t stand being so submissive anymore. I can’t live without using vulgar language - thank you for what? Thank? Shenny Marlugbee?! How many years has it been? Ten years have passed! The Japanese devils have all retired from Lugou Bridge and returned to their fields to plant Echigo rice! The relevant organizations you put the Chinese people... http://m.weibo.cn/client/version
	HejiuTsuruku	发布了头条文章：《【强塞安利/硬科普】为什么不只是女生需要 HPV 疫苗》 http://t.cn/Rt7FQhK
		Headline article published: ‘[Forced recommendation/Hard science popularization]Why not just girls need HPV vaccine’ http://t.cn/Rt7FQhK

Supplementary Table S14. Representative examples of popular tweets from super-spreaders in joy spreading networks.

Time	User	Tweet
2024	Henan Campus	这个开学季，我们来点不一样~#HPV 预防健康爆改 PK 赛#来了，每位女大赶快看过来，学姐喊你添加健康装备啦～#HPV 早知晓#要记得在未发生性生活的女性中接种 HPV 疫苗，将获得最佳预防效果；但对于已发生性行为的女性，研究实验表明接种 HPV 疫苗也有很好的保护作用，跟着学姐准没错，一起预防 HPV 感染，健康不掉队脆皮不破防…
		This school season, let's be different~#HPV Prevention Health Extreme Makeover PK Competition # is here, every female college student, hurry up and take a look, senior sisters call you to add health equipment~# HPV Early Awareness # Remember to get the HPV vaccine among women who have not had sex life, you will get the best prevention effect; But for women who have already engaged in sex life, research experiments have shown that getting the HPV vaccine also has a good protective effect. It is definitely not wrong to follow senior sisters to prevent HPV infection together. Not falling behind in the health team, and the brittle skin will not break...
	Tianjin Campus Headlines	这个开学季，跟着学姐一起爆改，加入#HPV 预防健康爆改 PK 赛# 无论什么计划，从最小行动开始做起，我们一起拆解拖延症！ HPV 感染开学早预防脆皮新生不破防#健康计划更不能拖延，快来 get 预防 HPV 感染的科学秘籍吧！一起为大学生活加 buff！#HPV 早知晓#戳链接了解更多精彩内容脆皮女大当自强！12 位知名博主喊你来入伙！…
		This school season, follow senior sisters to make explosive changes, join the # HPV Prevention and Health Extreme Makeover PK Competition# No matter what plan you have, start with the smallest actions and let's break down procrastination together! #Early prevention of HPV infection at the beginning of the school year is crucial for brittle new students. Health plans should not be delayed. Come and get the scientific tips for preventing HPV infection! Let's add buffs to college life together! #HPV early knowledge# Click on the link to learn more exciting content Brittle college girls should Strengthen Herself! 12 well-known bloggers are calling for you to join us...
	Xixiaotang_HipHop	#于贞 女王# 于贞的脱口秀全程！节目效果拉满！[doge]"我唱过某个品牌的 hpv 宣传曲，我很荣幸可以给大家宣传一些疫苗知识，但说实话，我觉得我们有些男 rapper 才是 HPV 传播大使。我是说他们歌太火了，堪称病毒式传播" "Rapper 一般塌方塌男不塌女，男 rapper 身上真有点东西" #痞老板 乙游 Freestyle#
		#Queen Yu Zhen# The entire talk show of Yu Zhen! Full program effect! [doge]"I have sung a brand's HPV promotional song before, and I am honored to spread some knowledge about HPV vaccines to everyone, but I think some male rappers are the true ambassadors for HPV transmission. I mean their songs are so popular, and they can be called viral spread. The public persona of Chinese male rappers is more easily ruined than that of female rappers, and male rappers really have something on their bodies."# Plaoban Otome game Freestyle #
2016	Zhuangshilaiyifama	【我的香港 hpv 疫苗以及游玩之旅】之前我做了一个 hpv 和宫颈癌的科普（科普内容是 http://weibo.com/1743308797/DsWVrFfDj 不懂 hpv 是什么的可以点链接进去看）然后我也就预约去打疫苗了，顺便玩了几天…
		[My Hong Kong HPV Vaccine and Travel Itinerary]<br>Previously, I did a science popularization post about HPV and cervical cancer (the content of the science popularization was: http://weibo.com/1743308797/DsWVrFfDj you can click the link to learn more if you don't know what HPV is). After that, I made an appointment to get vaccinated and played for a few days...
	CCTV News	【我国批准预防宫颈癌 HPV 疫苗上市 转给你关心的她！】国家食药监总局近日批准葛兰素史克（GSK）公司的预防用宫颈癌疫苗的进口注册申请。疫苗问世前，预防宫颈癌的主要手段是通过宫颈筛查。该疫苗的批准为我国宫颈癌的预防提供了新有效手段。但即使接种了宫颈癌疫苗仍然应定期筛查。（央视记者余静英）
		[China Approves HPV Vaccine for Cervical Cancer Prevention on Sale - Retweet it to the Ones You Care About!]<br>The State Food and Drug Administration recently approved the import registration

		application for the cervical cancer prevention vaccine produced by GlaxoSmithKline (GSK). Before the vaccine was introduced, the main method for preventing cervical cancer was through cervical screening. The approval of this vaccine provides a new and effective means for cervical cancer prevention in China. However, even after receiving the cervical cancer vaccine, regular screening should still be conducted. (CCTV reporter Yu Jingying)

Supplementary Table S15. Representative examples of popular tweets from super-spreaders in fear spreading networks.

Time	User	Tweet
2024	Yutouweibo	慎重接种 HPV 疫苗，洁身自好，保护好身体！#官方回应将 HPV 疫苗纳入免疫规划##国家卫健委回应能否免费接种 HPV 疫苗#，佳达修宫颈癌疫苗在二期和三期安全及有效性测试的负责人哈珀博士表示 a.不赞成（学校）小孩子接种疫苗，报道采访了疑似受害者的家属，并援引了 HPV 疫苗的疑似不良反应报告数量为 8228 宗，远远高于其他疫苗…
		Be cautious when getting the HPV vaccine, practice sexual discipline, and take care of your body! #Official Response on Including HPV Vaccine in Immunization Program# #National Health Commission's Response on Free HPV Vaccine# Dr. Harper, who was in charge of the Phase II and III safety and efficacy tests for the Gardasil cervical cancer vaccine, stated that (a) he is not in favor of (school) children receiving the vaccine. The report interviewed the families of suspected victims and cited that the number of suspected adverse reaction reports for the HPV vaccine is as high as 8228 cases, which is much higher than other vaccines...
	Sichuan Prison	【收藏！10 大高发癌症如何筛查预防】世界卫生组织认为癌症是一种生活方式疾病，三分之一的癌症完全可以预防。哪些因素易诱发癌症？肿瘤早期 5 个报警信号有哪些？远离癌症这些你应知道↓↓为自己和家人的健康，转存了解！
		[Favorites! How to Screen and Prevent the Top 10 High-Incidence Cancers] The World Health Organization believes that cancer is a lifestyle disease, and one-third of cancers can be completely prevented. What factors are prone to trigger cancer? What are the 5 early warning signals for tumors? Stay away from cancer—here's what you should know ↓↓ For your own and your family's health, retweet and favorite to learn more!
2016	Finance Net	【时隔十年 宫颈癌疫苗今日获准中国上市】18 日,葛兰素史克称,其\"希瑞适®\"获得国家食药监上市许可，成为国内首个获批用于预防宫颈癌的 HPV 疫苗。时隔十年已在全球百余个国家实施广泛接种的 HPV 疫苗终于进入中国市场。宫颈癌是中国 15 岁至 44 岁女性中的第二大高发癌症。全球平均每 2 分钟就有 1 女性死于宫颈癌... http://m.weibo.cn/client/version
		[The Cervical Cancer Vaccine Approved for Sale in China today After a Decade] On the 18th, GlaxoSmithKline announced that its \Cervarix®\ has obtained marketing authorization from the State Food and Drug Administration, becoming the first HPV vaccine approved for cervical cancer prevention in China. The HPV vaccine, which has been widely used in over a hundred countries around the world for a decade, has finally entered the Chinese market. Cervical cancer is the second most common cancer among women aged 15 to 44 in China. On average, on average, one woman dies from cervical cancer every two minutes worldwide... http://m.weibo.cn/client/version
	Zhihu	历经十年，用于预防宫颈癌的 HPV（人乳头瘤病毒）疫苗今日获准在中国上市。这意味着中国女性终于可以在正规医院，通过注射疫苗的方式远离宫颈癌——这一目前侵扰女性最致命和最凶险的癌症。关于接受宫颈癌疫苗注射有哪些注意事项？可以看看知乎上的讨论：http://t.cn/Rt79HMY（想看更多？下载知乎 App：... http://m.weibo.cn/client/version
		After a decade, the HPV (Human Papillomavirus) vaccine for cervical cancer prevention has been approved for sale in China today. This means that Chinese women can finally get vaccinated at public hospitals to protect themselves from cervical cancer—one of the most deadly and dangerous cancers currently affecting women. For those who want to know more about the precautions for receiving the cervical cancer vaccine injections? You can check out the discussions on Zhihu: http://t.cn/Rt79HMY (Want to see more? Download the Zhihu app: http://m.weibo.cn/client/version)

Supplementary Table S16. Representative examples of popular tweets from super-spreaders in sadness spreading networks.

Time	User	Tweet
2024	Doctor Chao Xu	许超医生我想咨询一下[允悲]今天我朋友跟我说她男朋友加了□的微信貌似，不知道有没有找□那个聊天记录删的干干净净，她说因为那个男的还长了疙瘩，还是长了痘痘啥的，就说自己去医院看过了是 hpv，是普通型病毒感染，用激光打掉了，我们想问，普通病毒感染要用激光打掉吗[泪][泪]真的不了解这个睡眠不足真的很糟糕…
		Dr. Chao Xu, I'd like to ask you something [facepalm]. Today, my friend told me that her boyfriend added a [sex worker] on WeChat. It seems like he might have chatted with her, but the chat records have been deleted. She said the guy had some bumps or something like acne, and he claimed that he went to the hospital and was diagnosed with HPV, a common type of viral infection, which was removed by laser. We want to ask, does a common virus infection need to be removed with a laser? [tearful][tearful] I really don't know much about this. Lack of sleep is really bad...
	Yezhini	内容仅供参考，如有不适请线下就医。但是 hpv 检查要一个星期出结果，我对象就想一起去检了，一起等结果，跑了两个医院都不给检，这种就很无语，我跟着去男医生就你这样说的，后来让他找，后面我爸又找了一个又是子宫癌难道是我爸携带…
		The content is for reference only. If you feel unwell, please seek medical attention offline. However, the HPV test takes a week to get the results. My partner and I wanted to get tested together and wait for the results together. But after visiting two hospitals, they both refused to conduct the test. It's really frustrating. When I went with him to see the male doctor, I explained the situation as I just did. Later, he was told to find another option. Then my dad tried to help and found another hospital, but they mentioned uterine cancer. Could it be that my dad is the carrier...
2016	Nikkei Business Publications, Inc	【宫颈癌疫苗】7 月 18 日多家媒体报道葛兰素史克称中国食药监局批准其宫颈癌疫苗（HPV 疫苗）上市的消息。HPV 疫苗在日本曾经由政府推荐接种过，但由于接到大量严重的副作用报告而被取消推荐资格改为自愿接种。日本在 2013 年 4 月开始由日本厚生省推荐给全国中学生以上的女性接种，结果一个月后就接到严重的副... http://m.weibo.cn/client/version
		[Cervical Cancer Vaccine] On July 18th, multiple media outlets reported that GlaxoSmithKline announced that the State Food and Drug Administration had approved its cervical cancer vaccine (HPV vaccine) for sale. The HPV vaccine was once recommended for vaccination by the Japanese government, but it was later removed from the recommendation list and changed to voluntary vaccination due to a large number of serious side effect reports. In April 2013, Japan's Ministry of Health, Labour and Welfare began recommending the vaccine to female students above middle school level nationwide, but severe adverse reactions were reported within a month... http://m.weibo.cn/client/version
	The Beijing News	【时隔十年 宫颈癌疫苗获准在国内上市】接种宫颈癌疫苗再也不用奔赴香港或者国外了。葛兰素史克(GSK)公司今日宣布，希瑞适(人乳头状瘤病毒(HPV)疫苗[16 型和 18 型])获得中国食品药品监督管理总局的上市许可，成为国内首个获批的预防宫颈癌的 HPV 疫苗。希瑞适在中国注册用于 9 到 25 岁女性的接种，采用 3 剂免疫... http://m.weibo.cn/client/version
		[Cervical Cancer Vaccine Approved for Sale in China After a Decade] There's no need to travel to Hong Kong or abroad for the cervical cancer vaccine anymore. GlaxoSmithKline (GSK) announced today that Cervarix (Human Papillomavirus (HPV) Vaccine [Types 16 and 18]) has obtained the marketing authorization from the State Food and Drug Administration, becoming the first HPV vaccine approved for cervical cancer prevention in China. Cervarix is registered in China for vaccination in females aged 9 to 25, with a 3-dose immunization... http://m.weibo.cn/client/version

Supplementary Table S17. Representative examples of popular tweets from super-spreaders in disgust spreading networks.

Time	User	Tweet
2024	Ms. Laoyao	妈的我都 34 岁肯定不是处女，妇科检查是一定要做的，之前在北京的时候，医生问你有没有结婚，我都老实说没有结婚，对方就会委婉问一下有过伴侣哈，没有 hpv，问我之前有没有做 hpv，我说没，她让我去找前台加一个，好了，结果为了做个妇科检查，我跑来跑去一早上，找了一堆人，找了两个主任签字，才总算把这个妇科检查给做上了…
		Damn, I'm already 34 years old and definitely not a virgin. Gynecological exams are a must. When I was in Beijing, the doctor asked if I was married, and I honestly said no. Then the other party would delicately ask if I had ever had a partner. I don't have HPV. When she asked if I had ever had an HPV test before. I said no, and she asked me to go to the front desk to add it. Well, in order to get a gynecological exam, I ran around all morning, talked to a bunch of people, and got signatures from two department heads before finally getting this gynecological examination done...
	Galaxy Ferry	女人感染 hpv 绝大多数都是被男人传染的啊你不知道吗，到处都是现成的例子，因为女性是纳入式天生就更容易感染，可以去大陆外打啊，我相信真正尊重伴侣的男人都会这样做吧#HPV 疫苗卖不动了#，倒也不会吧，不过我朋友确实跟我科普过九价和二价四价的区别，九价之前炒得那么火热，实际上管的年限好像很短…
		Don't you know that the vast majority of women who get infected with HPV are infected by men? There are ready-made examples everywhere. Women are more susceptible to infection by nature because of the receptive nature of their anatomy. They could get vaccinated outside the mainland. I believe men who truly respect their partners would do that, right? #HPV Vaccine Unsold#. I don't think that's true, but my friend did explain to me the difference between the 9-valent, bivalent, and quadrivalent vaccines. The 9-valent vaccine was so hyped before, but it seems like it actually covers a relatively short period of protection...
2016	PUMC_ Tanxianjie	发布了头条文章：《人乳头瘤病毒 HPV 疫苗终于在中国上市了，这 18 个问题您知道吗？》 http://t.cn/RtzUxcI
		Published a headline article: "The Human Papillomavirus (HPV) Vaccine is Finally Available in China—Do You Know These 18 Questions?" http://t.cn/RtzUxcI
	Guokr Net	【HPV 疫苗中国上市，它适合哪些人群接种？】据新京报，葛兰素史克公司今日宣布，希瑞适（HPV 疫苗）获得了中国食品药品监督管理总局的上市许可，这也是国内首个获批的预防宫颈癌的 HPV 疫苗。HPV 疫苗到底是做什么用的？适合什么样的人群接种？请看：http://t.cn/RP9uEe0
		[HPV Vaccine on Sale in China: Which Population is Suitable for Vaccination?] According to the Beijing News, GlaxoSmithKline (GSK) announced today that Cervarix (the HPV vaccine) has obtained the marketing authorization from the State Food and Drug Administration. This is also the first HPV vaccine approved in China for the preventing cervical cancer. What exactly is the HPV vaccine used for? What kind of population is suitable for vaccination? For more information, please see: http://t.cn/RP9uEe0

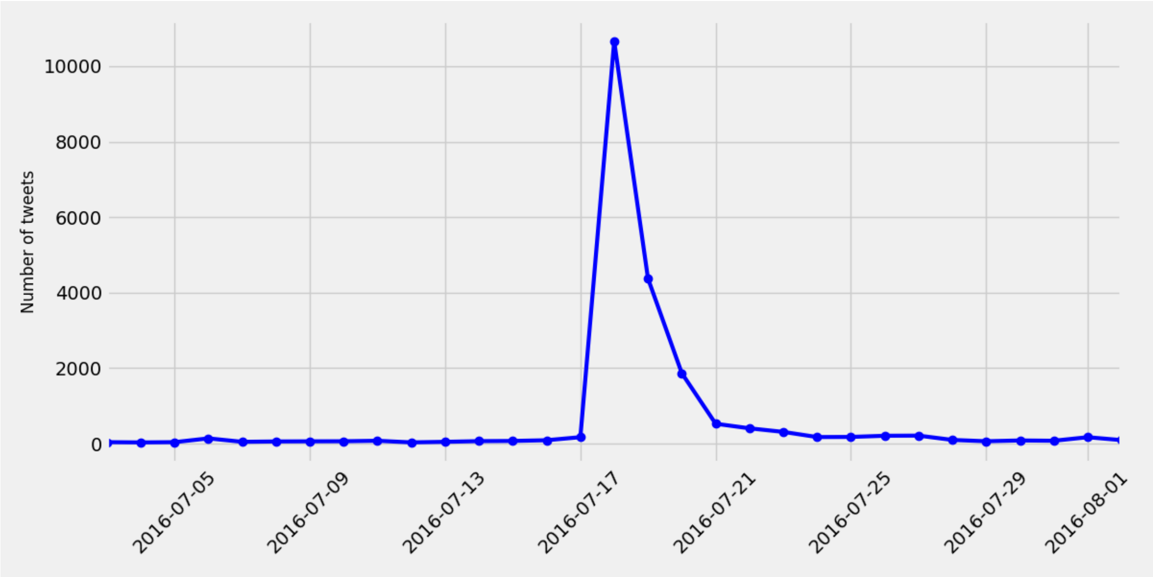

Supplementary Figure S1. Number of HPV-related tweets per day in 2016.

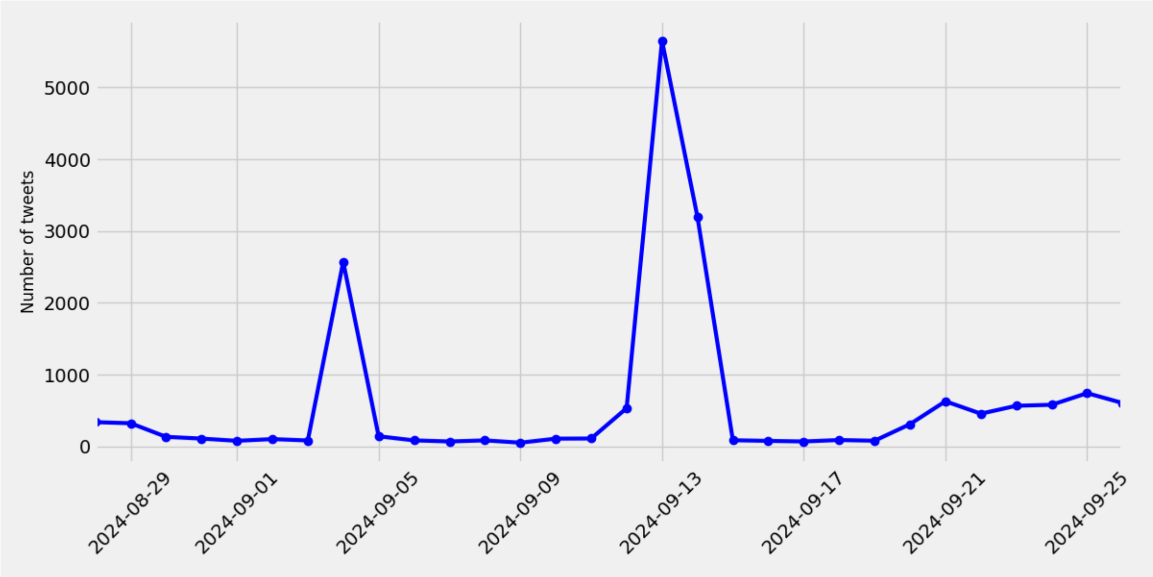

Supplementary Figure S2. Number of HPV-related tweets per day in 2024.

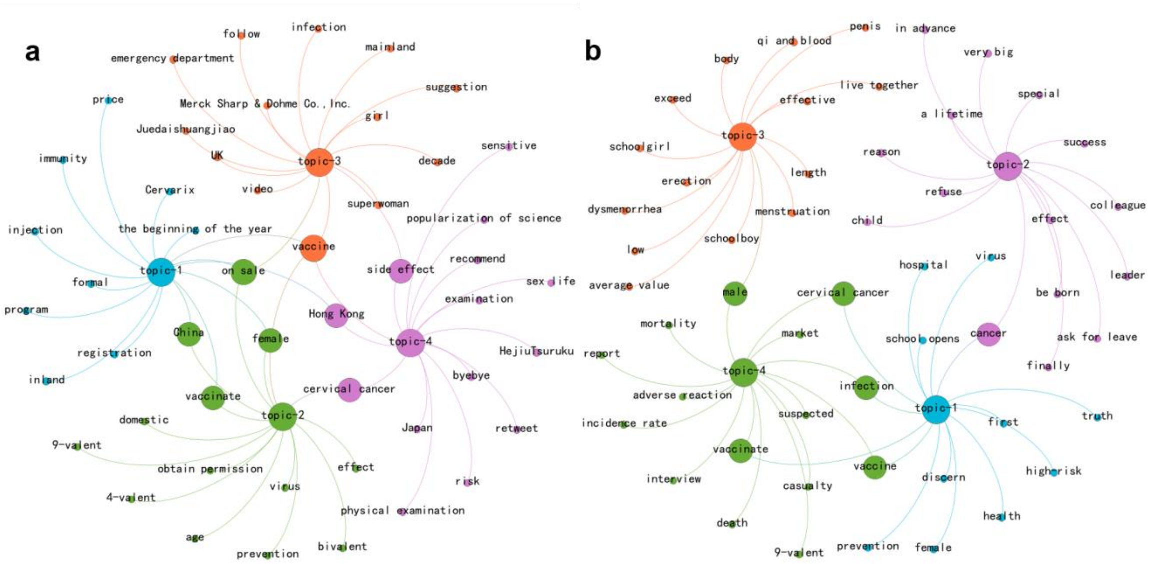

Supplementary Figure S3. Thematic network visualization of fear discussions. Note that sub-graphs (a) and (b) represent 2016 and 2024, respectively.

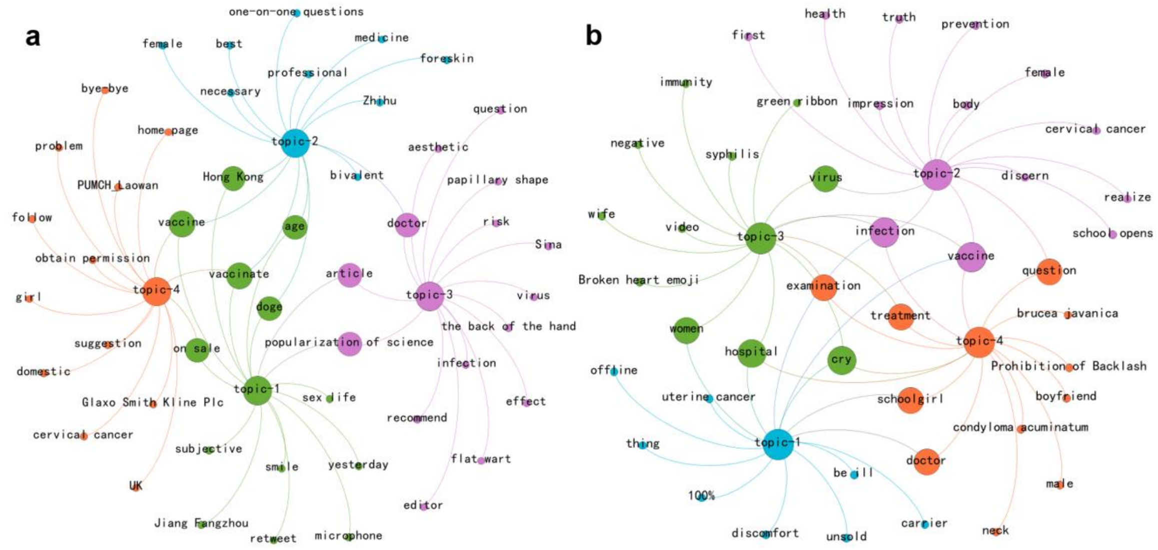

Supplementary Figure S4. Thematic network visualization of sadness discussions. Note that sub-graphs (a) and (b) represent 2016 and 2024, respectively.

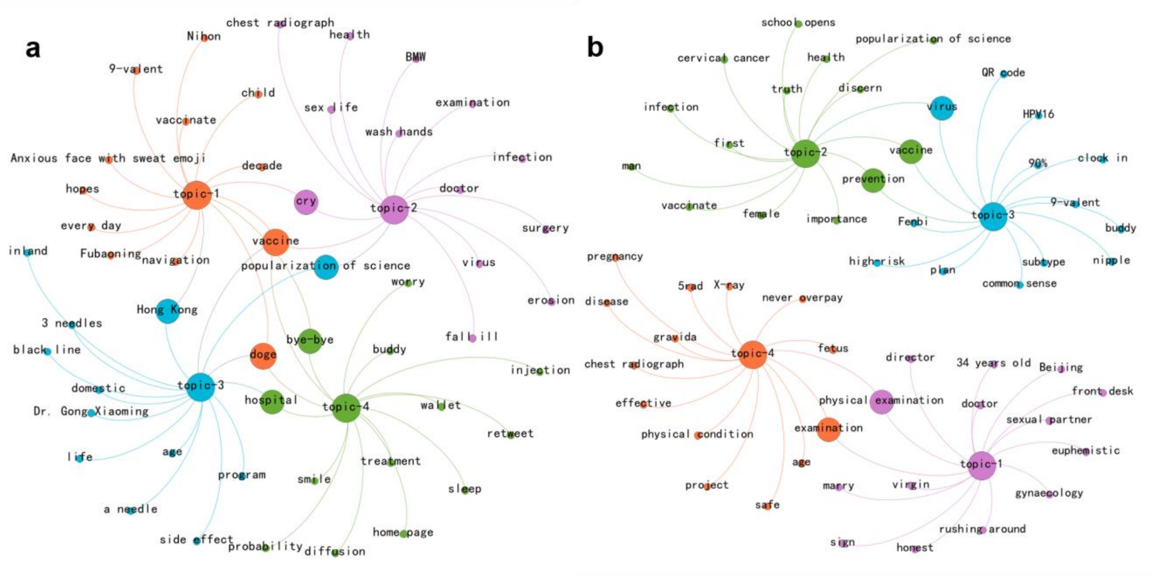

Supplementary Figure S5. Thematic network visualization of disgust discussions. Note that sub-graphs (a) and (b) represent 2016 and 2024, respectively.

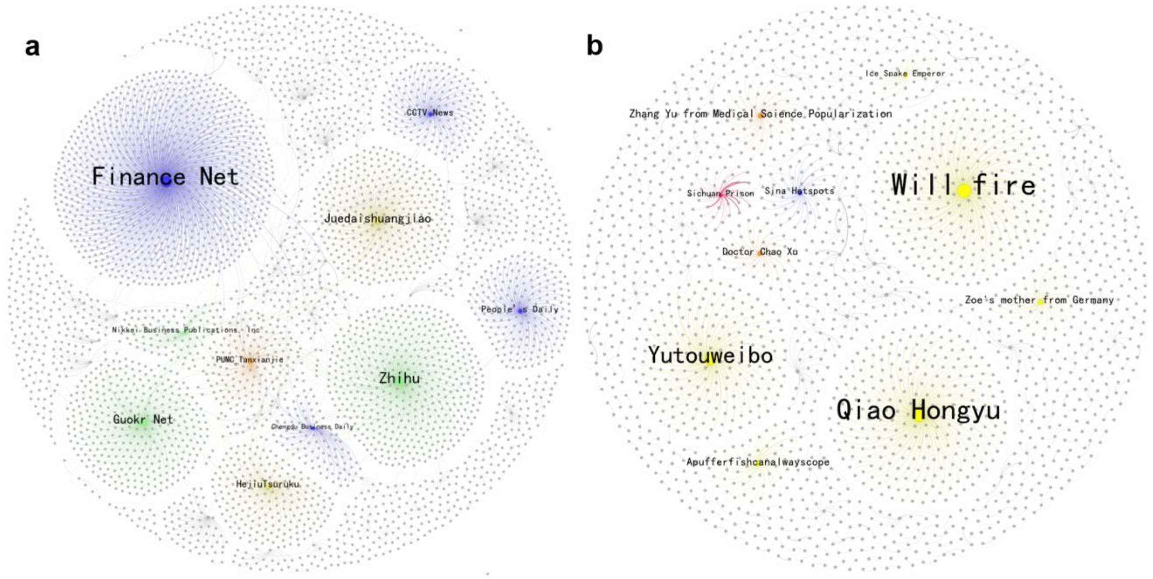

Supplementary Figure S6. The network of fear spreading. Note that sub-graphs (a) and (b) represent 2016 and 2024, respectively. The size of each node reflects its number of retweeters, and the top 10 super-spreaders are colored based on their verified type: blue for media, green for enterprises, red for the government, orange for celebrities, purple represents campuses and yellow for influential bloggers. The color of each edge matches that of its source node.

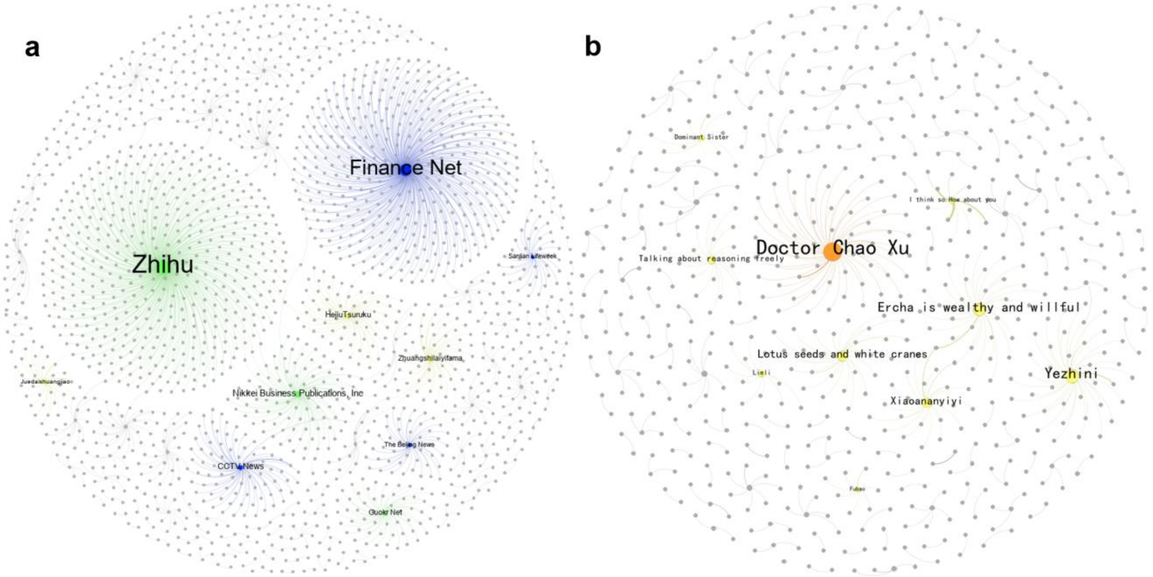

Supplementary Figure S7. The network of sadness spreading. Note that sub-graphs (a) and (b) represent 2016 and 2024, respectively. The size of each node reflects its number of retweeters, and the top 10 super-spreaders are colored based on their verified type: blue for media, green for enterprises, red for the government, orange for celebrities, purple represents campuses and yellow for influential bloggers. The color of each edge matches that of its source node.

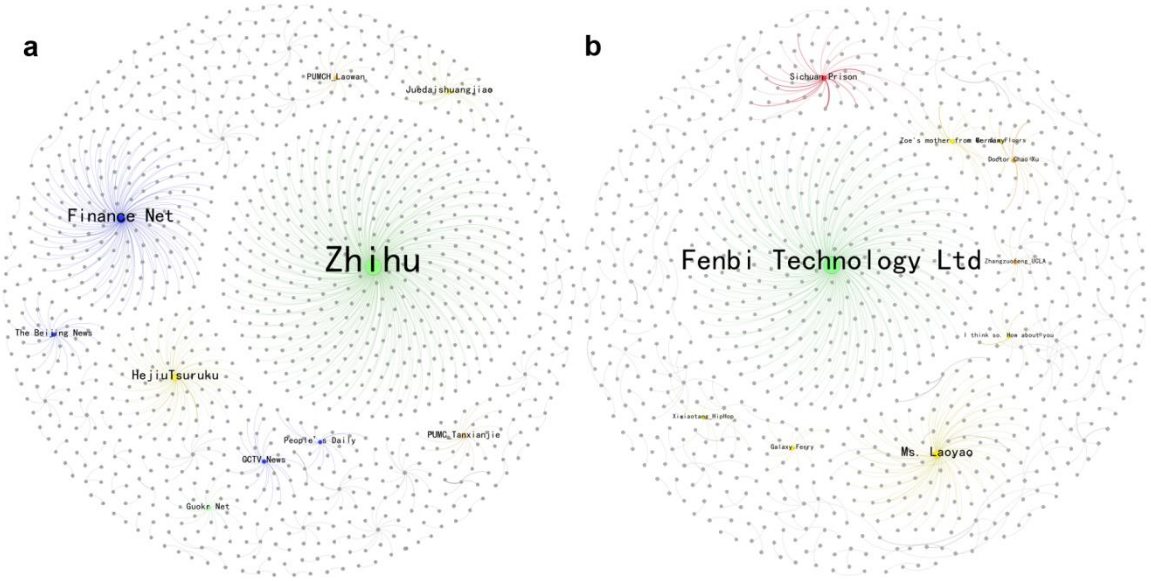

Supplementary Figure S8. The network of disgust spreading. Note that sub-graphs (a) and (b) represent 2016 and 2024, respectively. The size of each node reflects its number of retweeters, and the top 10 super-spreaders are colored based on their verified type: blue for media, green for enterprises, red for the government, orange for celebrities, purple represents campuses and yellow for influential bloggers. The color of each edge matches that of its source node.
